# Dysbiosis of Skin Microbiota in Psoriatic Patients: Co-occurrence of Fungal and Bacterial Communities

**DOI:** 10.3389/fmicb.2019.00438

**Published:** 2019-03-21

**Authors:** Zuzana Stehlikova, Martin Kostovcik, Klara Kostovcikova, Miloslav Kverka, Katerina Juzlova, Filip Rob, Jana Hercogova, Petr Bohac, Yishay Pinto, Atara Uzan, Omry Koren, Helena Tlaskalova-Hogenova, Zuzana Jiraskova Zakostelska

**Affiliations:** ^1^Institute of Microbiology, Czech Academy of Sciences, Prague, Czechia; ^2^BIOCEV, Institute of Microbiology, Academy of Sciences of the Czech Republic, Vestec, Czechia; ^3^Institute of Experimental Medicine, Czech Academy of Sciences, Prague, Czechia; ^4^Bulovka Hospital, Dermatovenerology Department, Second Faculty of Medicine, Charles University in Prague, Prague, Czechia; ^5^The Azrieli Faculty of Medicine, Bar-Ilan University, Safed, Israel

**Keywords:** psoriasis, microbiota, mycobiota, skin, sequencing

## Abstract

Psoriasis is a chronic inflammatory skin disease, whose pathogenesis involves dysregulated interplay among immune cells, keratinocytes and environmental triggers, including microbiota. Bacterial and fungal dysbiosis has been recently associated with several chronic immune-mediated diseases including psoriasis. In this comprehensive study, we investigated how different sampling sites and methods reflect the uncovered skin microbiota composition. After establishing the most suitable approach, we further examined correlations between bacteria and fungi on the psoriatic skin. We compared microbiota composition determined in the same sample by sequencing two distinct hypervariable regions of the 16S rRNA gene. We showed that using the V3V4 region led to higher species richness and evenness than using the V1V2 region. In particular, genera, such as *Staphylococcus* and *Micrococcus* were more abundant when using the V3V4 region, while *Planococcaceae*, on the other hand, were detected only by the V1V2 region. We performed a detailed analysis of skin microbiota composition of psoriatic lesions, unaffected psoriatic skin, and healthy control skin from the back and elbow. Only a few discriminative features were uncovered, mostly specific for the sampling site or method (swab, scraping, or biopsy). Swabs from psoriatic lesions on the back and the elbow were associated with increased abundance of *Brevibacterium* and *Kocuria palustris* and *Gordonia*, respectively. In the same samples from psoriatic lesions, we found a significantly higher abundance of the fungus *Malassezia restricta* on the back, while *Malassezia sympodialis* dominated the elbow mycobiota. In psoriatic elbow skin, we found significant correlation between occurrence of *Kocuria*, *Lactobacillus*, and *Streptococcus* with *Saccharomyces*, which was not observed in healthy skin. For the first time, we showed here a psoriasis-specific correlation between fungal and bacterial species, suggesting a link between competition for niche occupancy and psoriasis. However, it still remains to be elucidated whether observed microbial shift and specific inter-kingdom relationship pattern are of primary etiological significance or secondary to the disease.

## Introduction

The skin is our major interface with the outside environment. It harbors diverse site-specific microbial communities consisting of bacteria, fungi, and viruses (Grice and Segre, 2011). The skin microbiota protects against harmful microbes, maintains skin homeostasis and educates our immune system (Gallo and Nakatsuji, 2011; Belkaid and Naik, 2013; Grice, 2015). It has been shown that healthy microbiota can enhance the skin’s protective barrier and strengthen the immune response of keratinocytes by inducing a higher expression of antimicrobial peptides and formation of biofilms (Wanke et al., 2011). On the other hand, microbial dysbiosis could cause or exacerbate skin diseases (Cogen et al., 2008; Gallo and Nakatsuji, 2011; Grice and Segre, 2011).

Psoriasis is a chronic inflammatory skin disease that involves a dysregulated interplay among immune cells, keratinocytes and environmental triggers, including microbiota (Nestle et al., 2009). Currently, psoriasis is perceived as a complex systemic immune mediated disease or syndrome, significantly associated with many chronic diseases including arthritis, heart disease, diabetes, metabolic syndrome, inflammatory bowel disease (IBD), or celiac disease (Sundarrajan and Arumugam, 2016; Singh et al., 2017). Pathogenesis of some of the aforementioned diseases, e.g., rheumatoid arthritis, obesity or IBD, has been also connected to microbial shifts (Berthelot and Le Goff, 2010; Befus et al., 2015; Chu et al., 2016). Indeed, changes in gut and skin microbiome have been recognized as important triggers for initiation or progression of psoriasis in humans as well as in animal models of psoriasis (Fry et al., 2013; Zanvit et al., 2015; Zakostelska et al., 2016; Drago et al., 2018). Moreover, changes in microbiota composition could be a one of the factors that leads to disruption of intestinal barrier function in psoriatic patients (Mattozzi et al., 2012; Yan et al., 2017).

Current studies describing the differences in microbiota composition in psoriatic patients suffer from inconsistent approaches, such as choosing different sampling techniques, different variable regions of the 16S rRNA gene and different skin sampling sites (Gao et al., 2008; Fahlen et al., 2012; Alekseyenko et al., 2013; Tett et al., 2017). Moreover, some of them are focused on microbiota composition, while others describe function and try to find connective features with disease state (Alekseyenko et al., 2013; Yan et al., 2017). The results differ in species richness (alpha diversity) and between sample diversity (beta) (Gao et al., 2008; Fahlen et al., 2012; Alekseyenko et al., 2013; Tett et al., 2017; Yan et al., 2017). Analyses of microbiota composition from swabs and biopsy samples revealed Firmicutes as the most dominant phylum in psoriatic lesions (Gao et al., 2008; Fahlen et al., 2012). In-depth analyses of microbiota composition in psoriatic patients compared to healthy controls revealed an increased abundance of the genus *Streptococcus* and an underrepresentation of the genus *Propionibacterium*, while presenting inconsistent findings on the abundance of *Staphylococcus* (Gao et al., 2008; Fahlen et al., 2012; Alekseyenko et al., 2013; Tett et al., 2017). To our knowledge, none of the published studies concerning the composition of mycobiome in patients with psoriasis used next generation sequencing and most of them focused mainly on *Malassezia* (Paulino et al., 2008; Jagielski et al., 2014; Takemoto et al., 2015). The most extensive study by Takemoto et al. (2015) described a higher fungal diversity and overall lower abundance of *Malassezia* in psoriatic lesions. Since no bacterial or fungal microbiota components have been robustly identified as being associated with psoriasis across the studies, new comparable standardized studies are necessary to extend the existing data on microbiome composition and function.

Here, we conduct a comprehensive study mapping the overall composition of bacterial and fungal communities in psoriatic lesions, unaffected psoriatic skin and healthy skin. We compare two typical sites of psoriasis incidence – the elbow and the back. Moreover, we compare three different sampling methods, namely swabs, scrapings and biopsies, since each of these techniques produces slightly different results of microbial composition.

## Materials and Methods

### Patients and Sample Collection

Patients diagnosed with chronic plaque psoriasis were recruited at the Department of Dermatovenereology, Bulovka Hospital (Czechia). Altogether, 34 patients with chronic plaque psoriasis (6 females and 28 males) and 25 healthy controls (14 females and 13 males) were recruited. The average age ± standard deviation of patients was 45 ± 12 years and 44 ± 13.3 for healthy controls. Body Mass Index (BMI) was 20.6 ± 5.9, Psoriasis Area Severity Index (PASI) of psoriatic patients was 6 ± 7, and Physician Global Assessment (PGA) was 2 ± 1. Patients were clinically classified and their medical history was recorded. The majority of tested patients were under various treatment protocols depending on disease severity. However, seven psoriatic patients without prior treatment were included in our study. Majority of the study cohort, patients (85%) and controls (92%) alike, are residents of town municipalities. Characteristics and medical history of patients and healthy controls are summarized in Supplementary Tables S1, S2. The study was approved by The Ethical Committee of Bulovka Hospital with approval number 28.7.2014/7292/EK (Czech Republic) and all participants signed informed consent forms.

Sampling was performed by an accredited dermatologist using a protocol from the Human Microbiome Project Consortium [HMPC] (2012b) to minimize collection bias. Swabs, scrapings, and punch biopsy samples were taken either from the dorsal (back) or olecranon (elbow) skin areas. In psoriatic patients, both psoriatic and contralateral unaffected sites were sampled. Altogether 68 swabs, 68 scrapings, and 19 biopsies from psoriatic patients and 25 swabs, 23 scrapings, and 8 biopsies from healthy controls were analyzed (Supplementary Table S3). Briefly, swab samples were taken from a 2 × 2 cm area using flocked swabs (FLOQSwabs^TM^ COPAN Diagnostics Inc., United States), soaked in sterile SCF-1 buffer [50 mM Tris buffer (pH 7.6), 1 mM EDTA (pH 8.0), 0.5% Tween 20] (Human Microbiome Project Consortium [HMPC], 2012b). Scraping samples were obtained from a 2 × 2 cm area using a scalpel similarly as previously described (Grice et al., 2008). Scraping samples were collected using a flocked swab soaked in sterile SCF-1 buffer. Samples were stored in 400 μl of SCF-1 buffer. Biopsies from psoriatic patients were taken at around 2 mm of size using a biopsy punch and stored dry (Fahlen et al., 2012). Biopsies from healthy controls were taken from the terminal end of elliptical specimens from patients undergoing wide excision of a birthmark. During biopsy sampling, the same local anesthetic (4% supracain) was used in both groups. No antiseptic treatment was used before sampling. All swabs, scrapings and biopsy samples were immediately frozen at −80°C.

### DNA Extraction

Extraction of total DNA from swabs and scrapings was performed using DNeasy PowerBiofilm Kit (Qiagen, Germany) with minor changes in the protocol. Thawed swabs and scraping samples were thoroughly vortexed. Nipped off swabs were aseptically removed and samples were centrifuged for 10 min at 13,000 × *g*. The precipitate was diluted in 350 μl of the first kit buffer (MBL), and 100 μl of the second kit buffer (FB) was added to the sample, followed by homogenization at maximum speed (6.5 m/s) for 1 min (Fast Prep, MP Biomedicals, United States). Incubation of the bead tubes at 65°C for 5 min was excluded from the protocol as it resulted in a low DNA yield. The remaining steps were performed as per manufacturer’s instructions. Total DNA from punch biopsies was extracted by MasterPure^TM^ Complete DNA and RNA Purification Kit (Epicentre, Madison, WI, United States). Biopsies were homogenized at maximum speed (6.5 m/s) for 1 min in a solution of 300 μl Tissue and Cell Lysis Solution and 1 μl of Proteinase K (Fast Prep, MP Biomedicals, United States). Further isolation was performed following manufacturer’s instructions.

### PCR Amplification

To compare sequencing of different variable regions of the 16S rRNA gene (V1V2 and V3V4), PCR amplification of the V1V2 region was carried out with the 27F (5′-AATGATACGGCGACCACCGAGATCTACACGTACGTACGG TAGAGTTTGATCCTGGCTCAG-3′) and the 338R (5′-CAAGCAGAAGACGGCATACGAGATCGCTCACAGAATCC ACACTCATCATGCTGCCTCCCGTAGGAGT-3′) primers, already including Illumina adaptors and unique 12-nucleotide barcode on 338R (Hamady et al., 2008). The PCR reaction mixture consisted of 1X PrimeStar Max DNA polymerase (Takara Bio Co., Shiga, Japan), 0.3 μM primers and approximately 20 ng of DNA in 50 μl reaction (Koren et al., 2011). Cycle parameters were 33 cycles of denaturation (98°C, 10 s), annealing (55°C, 5 s) and extension (72°C, 5 s), and a final elongation at 72°C for 2 min. Microbial community profiling using the V3V4 region for bacteria and the ITS1 region for fungi was performed using 341F (5′-CCTACGGGNGGCWGCAG-3′) and 806R (5′-GGACTACHVGGGTWTCTAAT-3′) degenerate bacterial primers, and using ITS1-5.8Sfw (5′-AAGTTCAAAGAYTCGATGATTCAC-3′) and ITS1-5.8Srv (5′-AAGTTCAAAGAYTCGATGATTCAC-3′) degenerate fungal primers, which were all barcoded to enable multiplexing of sequencing libraries. Duplicates containing 25 μl of the reaction mixture were prepared for each sample. PCR amplification was carried out with 1X PrimeStar Max DNA polymerase (Takara Bio Co., Shiga, Japan), 0.4 μM primers and approximately 10 ng of template as the final concentrations. Thermal cycling parameters were 35 cycles of denaturation (94°C, 3 min), annealing (55°C, 5 s) and extension (72°C, 10 s), with a final extension at 72°C for 2 min. Replicate PCR products were pooled to minimize random PCR bias and the length of PCR products was determined using agarose gel electrophoresis. Extraction controls and no-template control were processed similarly.

### Sequencing, Classification, and Data Analysis

V1V2 PCR products were purified using AMPure magnetic beads (Beckman Coulter, United States) and quantified with a Quant-iT^TM^ PicoGreen^TM^ dsDNA Assay Kit (Thermo Fisher Scientific, United States). Samples were equally pooled at a concentration of 30ng/μl, loaded on 2% E-Gel (Thermo Fisher Scientific, United States) and purified with NucleoSpin Gel and PCR Clean-up (Macherey-Nagel, Germany). Purified products were sequenced on the Illumina MiSeq platform (Genomic Center, Faculty of Medicine, BIU, Israel). V3V4 amplicons were plate-purified using a SequalPrep^TM^ Normalization Plate (96) Kit (Invitrogen). Equal amounts of the PCR product from each sample were pooled and MiSeq platform compatible adapters were ligated using KAPA HyperPlus Kit (Roche, United States). The library was quantified using KAPA Library Quantification Kit (Illumina, United States) and sequenced on the MiSeq platform using 2 × 300 bp kit at the CEITEC Genomics Core Facility (Brno, Czechia).

Sequencing data were processed using QIIME version 1.9.1 (Caporaso et al., 2010). The raw sequence data are available in the Sequence Read Archive (SRA) under the accession number SUB4321198.

To allow analysis of both datasets together, closed-reference operational taxonomic unit (OTU) picking was used due to non-overlapping regions employed (Navas-Molina et al., 2013; Rideout et al., 2014). Standard procedure involved quality filtering, chimera detection and removal and demultiplexing based on default criteria. Altogether, 522 individual amplicon libraries were found consisting of a total of 17.26 million paired-end reads (reads per sample after all filtering steps, including chimera and quality filtering, averaged 22,092). Read clustering was performed using a 97% identity threshold. Taxonomic classification was performed based on the bacterial 16S database GREENGENES 13.8 (DeSantis et al., 2006).

In the fungal dataset, 212 individual amplicon libraries consisted of a total of 1,799,822 paired-end reads (reads per sample after all filtering steps, including chimera and quality filtering and ITS extraction, averaged 2,351). Read clustering was performed using a 97% identity threshold against the UNITE database version 7.2 (Koljalg et al., 2013) and identification was conducted using RDP classifier up to the species level as in the case of bacterial datasets with default confidence value (Wang et al., 2007).

Alpha and beta diversity were calculated based on rarefied datasets (220 sequences for bacteria, 200 sequences for fungi per sample) to deal with unequal sequencing output per sample. Normalization was done on the level that was sufficient based on the rarefaction curves that were approaching plateau with selected number of reads. Several alpha diversity indices were calculated, including Chao1, Shannon, Gini-Simpson and observed species. Statistical significance was confirmed using Kruskal–Wallis test with Dunn’s multiple comparison test or Mann–Whitney test. Beta diversity was presented in principal coordinate analysis (PCoA) plots and assessed using several indices, including weighted and unweighted UniFrac distances for bacterial analysis, and Binary–Jaccard and Bray–Curtis metrics for fungal analysis. Statistical significance was confirmed using PERMANOVA. To identify the main differences in bacterial taxa between the V1V2 and V3V4 regions, we detected differential features using non-parametric factorial Kruskal–Wallis (KW) sum-rank test, compared between-group consistency using Wilcoxon rank-sum test and performed an LDA analysis to assess the effect size summarized using LEfSe (Linear discriminant analysis Effect Size) (Segata et al., 2011). Functional profiling of the recovered communities was done by PICRUST analysis (Langille et al., 2013). To determine the discriminative features for both taxonomic and metabolic profiles of communities, LEfSe analysis tool was employed (Segata et al., 2011). Predicted functional profiles were further analyzed with HUMAnN using KEGG orthology (Abubucker et al., 2012).

### Bacteria–Fungi Correlation

Relative abundances of bacteria and fungi were correlated in both back and elbow skin samples. Only fungi and bacteria present in at least one-third of the patients in any group of samples (psoriatic, unaffected, and healthy skin) were kept for further analysis. Pearson correlation coefficients and *p*-values were calculated for each bacterium–fungus pair and for each group of samples separately.

### Enzyme-Linked Immunosorbent Assay

Serum samples were taken from psoriatic patients (*n* = 28) and healthy controls (*n* = 27) and stored at −20°C until analysis. Serum level of intestinal fatty acid binding protein (I-FABP) was determined by commercially available ELISA kit (HBT, Hycult Biotech, Netherlands). Serum levels of caspase-cleaved cytokeratin 18 fragment (ccCK 18) and total cytokeratin 18 (CK18) were determined by commercially available ELISA kits M30 and M65, respectively (Apoptosense, Peviva, Sweden), and apoptotic index was counted as their ration (M30/M65). The concentration of ccCK18 and total CK18 in serum was detected in U/L. All assays were performed according to the manufacturer’s instructions.

## Results

### The V3V4 Region Captures Wider Microbial Diversity Than the V1V2 Region

**FIGURE 1:**
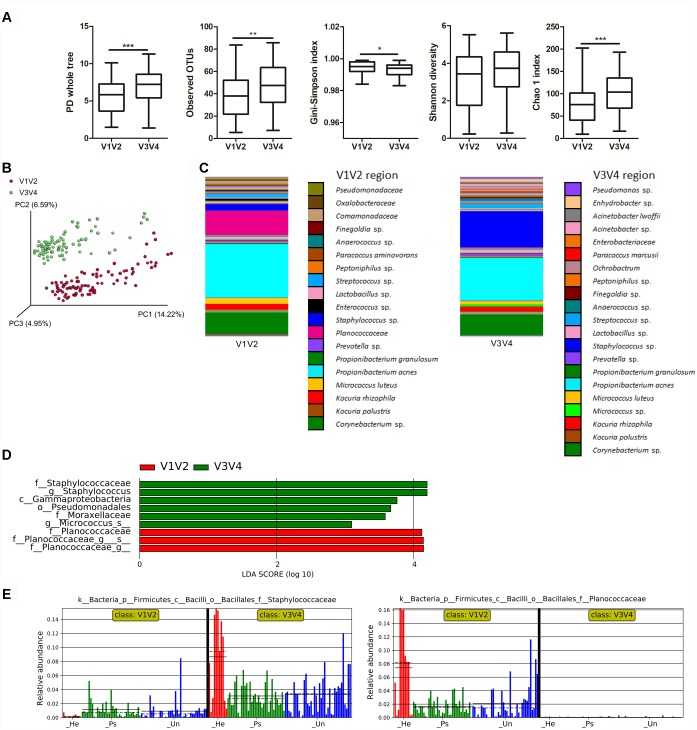
Differences in microbiota composition due to sequencing of different 16S rRNA regions. **(A)** Alpha diversity metrics for V1V2 and V3V4 regions are shown using PD whole tree, OTU richness, Gini-Simpson index, Shannon diversity index, and Chao1 index. **(B)** PCoA of Unweighted UniFrac distances (beta diversity) between the respective regions. Statistical significance was confirmed using PERMANOVA; *p* ≤ 0.001 Red: V1V2, Green: V3V4. **(C)** Relative abundances of major taxonomic groups in V1V2 and V3V4 regions. **(D)** LEfSe analysis describing the significant differences in genera between V1V2 (red) and V3V4 (green) regions. **(E)** Histogram of the Staphylococcaceae and Planococcaceae relative abundances in V1V2 and V3V4 regions. Disease status (Ps, psoriatic; Un, unaffected; He, healthy skin) is color-coded and the mean (solid line) and median (dashed line) are marked. ^∗^*p* < 0.05; ^∗∗^*p* < 0.01, and ^∗∗∗^*p* < 0.001.

To point out differences in observed bacterial diversity caused by sequencing of different 16S rRNA variable regions, we sequenced the V1V2 and V3V4 regions in skin samples from psoriatic patients and healthy controls. Identical skin samples obtained from the back were sequenced for both regions and were included in the analysis comparing V1V2 to V3V4 marker regions. Observed and estimated richness was significantly higher when using the V3V4 region compared to the V1V2 region (Figure 1A). There were no significant differences in Shannon diversity index and when using the Gini-Simpson index, a significant increase in V1V2 region was observed (Figure 1A). PCoA of unweighted UniFrac distances revealed a significant difference in beta diversity between the V1V2 and V3V4 regions (*p* ≤ 0.001) (Figure 1B). In addition, PCoA of weighted and unweighted UniFrac distances between the respective regions and type of sampling showed also significant changes (Supplementary Figure S1). Relative proportional differences in bacterial abundances between the V1V2 and V3V4 region are shown in Figure 1C. LEfSe analysis revealed the class Gammaproteobacteria, order Pseudomonadales, families Moraxellaceae and Staphylococcaceae, and genera *Staphylococcus* and *Micrococcus* as the most discriminatory for V3V4 region; for the V1V2 region it was the family Planococcaceae which was not detected at all using the V3V4 primer set (Figure 1D,E).

### Various Sampling Approaches Result in Similar Bacterial Diversity but Different Genera Abundance When Analyzing the V3V4 Region

To evaluate the relative diversity associated with each sampling approach (swab, scraping, and biopsy) in affected and unaffected skin of psoriatic patients and in healthy control skin, we calculated richness and evenness using several diversity indices to account for their specific biases. In psoriatic lesions on the back and elbow, we found that swabs and scrapings result in similar alpha diversity profiles, with emphasis on the consistency of species abundance and diversity. In unaffected back and elbow skin, the observed microbial diversity was similar in swabs and scrapings, in contrast to a slightly higher diversity in back biopsies. In healthy back and elbow skin, we observed similar alpha diversity measures. Weighted and unweighted UniFrac analysis showed significant clustering of back samples in diseased or unaffected psoriatic skin. In healthy back skin, significant changes were observed only when weighted PCoA analysis was applied (Supplementary Figures S2, S3). There was no clustering in beta diversity of diseased or unaffected psoriatic samples isolated from the elbow (Supplementary Figure S3). LEfSe analysis of back samples revealed several differentially abundant taxa in biopsies, scrapings and swabs. Elbow samples showed the most differentially abundant taxa in swabs. The main features revealed by LEfSe analysis of the V3V4 region are summarized in Table 1. Moreover, relative abundances of selected phylum and genus are displayed in Supplementary Table S4. Representative differences in bacterial abundance between sampling methods for the V1V2 region are summarized in Supplementary Table S5.

**Table 1:** The main discriminative bacterial features related to sampling approaches.

Representative bacterial biomarkers related to sampling approaches				
		Sample type		
Sample site		Swabs	Scrapings	Biopsies
Back	Psoriatic	*Peptoniphilus*	*Streptococcus*	Aeromonadaceae
			*Anaerococcus*	Microbacteriaceae
			*Veillonella*	*Lactobacillus*
				*Bacillus flexus*
				Lachnospiraceae
				*Allobaculum*
				*Parabacteroides*
				*Bacillus megaterium*
				*Acinetobacter guillouiae*
				*Corynebacterium durum*
				Mollicutes
	Unaffected	*Staphylococcus*	Moraxellaceae Comamonadaceae	Enterobacteriaceae *Lactobacillus Bacteroides*
				*Bacillus flexus*
				Clostridiales
				*Prevotella*
				*Parabacteroides distasonis*
	Healthy	*Finegoldia*	Micrococcaceae	Enterobacteriales
		*Peptoniphilus*	Xanthomonadales	*Facklamia*
				*Cloacibacterium*
				*Bacillus flexus*
				*Dermacoccus*
				*Parabacteroides distasonis*
				*Mycobacterium*
Elbow	Psoriatic	Tissierellaceae	*Pseudomonas*	N/A
		*Kocuria rhizophila*		
		*Kocuria palustris*		
	Unaffected	Ruminococcaceae	Enterobacteriaceae	N/A
	Healthy	*Chryseobacterium*	*Pseudomonas*	N/A

### Descriptive and Functional Analysis of Bacterial Microbiota Isolated From Swabs

**FIGURE 2:**
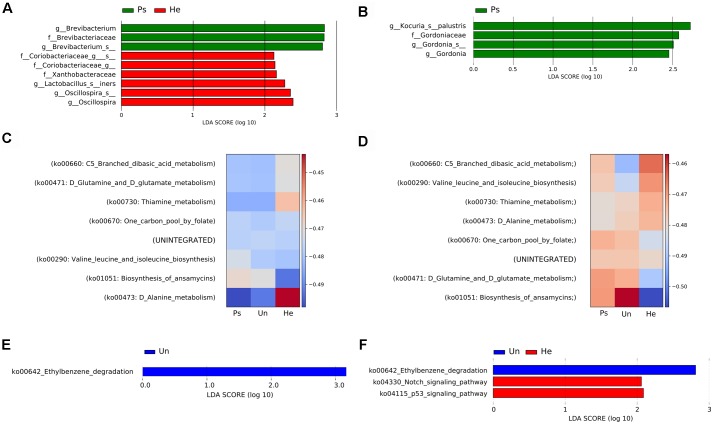
Analysis of bacterial communities isolated from swabs using the V3V4 region of the 16S rRNA gene. LEfSe analysis revealed microbial patterns that are significantly differentially abundant in back **(A)** or elbow **(B)** samples. Predicted functional profiles of metabolic pathways in back **(C)** or elbow **(D)** samples associated with psoriatic, unaffected or healthy control skin. Heat maps include only KEGG pathways with abundance above 1% for each site and disease status. The differences are color-coded by the relative abundance of the metabolic pathway. Red: high abundance; blue: low abundance. Discriminative metabolic pathways in back **(E)** or elbow **(F)** samples revealed by LEfSe analysis. Disease status: Ps, psoriatic; Un, unaffected; He, healthy skin.

For a more detailed analysis of the microbial composition related to sample site, we further proceeded with analysis of the V3V4 region in swab samples. This sampling approach yielded comparable richness and evenness to other sampling techniques. We did not observe any significant differences in overall microbial diversity or richness and evenness of microbial populations in either back or elbow samples (Supplementary Figure S3), but each sampling site had some taxa significantly associated with it. In back samples, we found *Brevibacterium* to be associated with psoriatic skin, and families Coriobacteriaceae and Xanthobacteraceae with healthy skin (Figure 2A). In elbow samples, we identified only the species *Kocuria palustris* and genus *Gordonia* as differentially abundant for psoriatic skin (Figure 2B). Next, we predicted functional profiles and gene content of bacterial community samples. Many metabolic pathways described in our analyses were similar, since the pathways were common to all samples. Heat map representation of the results from the HUMAnN analysis shows six metabolic pathways common for the back and the elbow: ko00670: One carbon pool by folate; ko00290: Valine leucine and isoleucine biosynthesis, ko00471: D-Glutamine and D-glutamate; ko00660: C5-Branched dibasic acid metabolism; ko00473: D-Alanine metabolism; ko01051: Biosynthesis of ansamycins. However, these discriminative pathways were represented with different abundance in the back (Figure 2C) and in the elbow (Figure 2D). We then used the LEfSE tool to segregate only those pathways that were discriminative for each site (back, elbow) and disease status (psoriatic, unaffected or healthy) (Segata et al., 2011). Using LEfSe, we showed that the pathway ko00642: Ethylbenzene degradation is significantly associated with unaffected skin both on the back (Figure 2E) and the elbow (Figure 2F), while only elbow samples showed further association with pathways ko04330: Notch signaling and ko04115: p53 signaling (Figure 2F). For descriptive and functional analysis of swab samples done by sequencing the V1V2 region, see Supplementary Figure S4.

### Psoriatic Back Skin Displays Increased Fungal Diversity With Higher Taxa Similarity Than Psoriatic Elbow Skin

Apart from the analysis of bacterial communities, we also characterized the skin fungal communities in order to detect possible interference of different niche competitors. Analysis of alpha diversity revealed significant differences in fungal richness in swabs from psoriatic and unaffected back skin (Chao1 index) but not in healthy controls. Gini-Simpson and Shannon diversity indices were both non-significant in all sampling approaches and all sampled sites, indicating more or less stable evenness of fungal taxa across back samples (Supplementary Figure S6). In elbow samples, all alpha diversity measures were non-significant, suggesting a similar fungal distribution regardless of the type or site of sampling (Supplementary Figure S7). Binary-Jaccard metrics revealed significantly different clustering in psoriatic back lesions for different sampling approaches (Supplementary Figure S6). Beta diversity in unaffected and healthy skin on the back and the elbow and in psoriatic lesions on the elbow remains unchanged (Supplementary Figures S5, S7). To uncover specific fungal biomarkers related to each sampling method to a greater extent, we applied LEfSe analysis focused on different sampling methods, i.e., swabs, scrapings, and biopsies. For better clarity, these results are summarized in Table 2.

**Table 2:** The main discriminative fungal features related to the sampling approaches.

Representative fungal biomarkers in psoriatic and healthy skin				
		Sample type		
Sample site		Swabs	Scrapings	Biopsies
Back	Psoriatic	*Malassezia*	None	*Vermiconia*
		Cystobasidiomycetes		*Venturia*
		*Cladosporium sphaerospermum*		*Cryptococcus*
				*Malassezia slooffiae*
				*Candida parapsilosis*
				*Candida tropicalis*
				*Cyberlindnera jadinii*
	Unaffected	None	*Intersonilia*	*Cephalotrichum*
				*Penicillium*
	Healthy	None	None	*Melampsora*
Elbow	Psoriatic	*Malassezia sympodialis*	*Malassezia slooffiae*	N/A
		*Penicillium*		
		Leotiomycetes		
	Unaffected	*Malassezia slooffiae*	Dothideomycetes	N/A
	Healthy	None	*Debaryomyces hansenii*	N/A
			*Saccharomyces cerevisiae*	
			*Penicillium miczynskii*	

### Descriptive and Functional Analysis of Fungal Microbiota Isolated From Swabs

**FIGURE 3:**
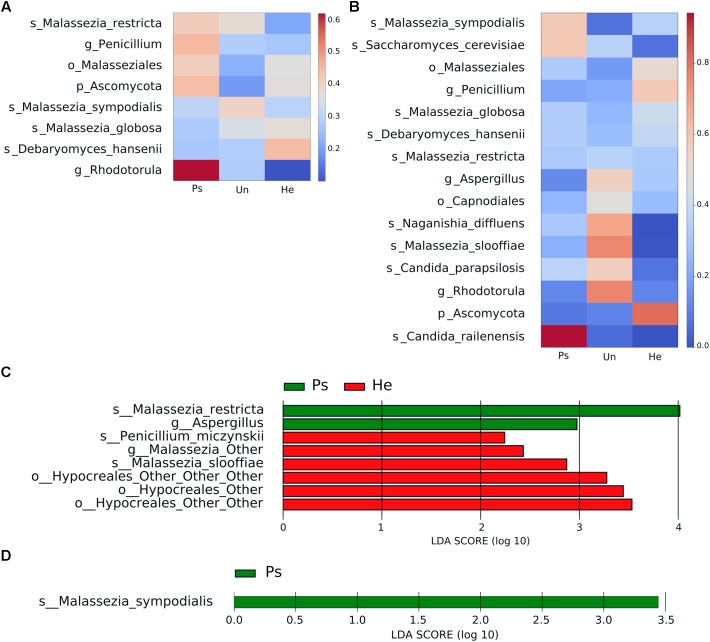
Distinctive fungal patterns of swab samples related to disease status. Heat maps depict relative bacterial abundance in back **(A)** and elbow **(B)** samples. The cut-off abundance was set at 1%. The differences are color-coded by the relative abundance of bacteria. Red: high abundance; blue: low abundance. LEfSe analysis showing discriminative bacterial patterns in back **(C)** and elbow **(D)** samples in psoriatic, unaffected psoriatic, or healthy skin. Disease status: Ps, psoriatic; Un, unaffected; He, healthy skin.

Following the bacterial analysis, we chose swabs as the most effective sampling approach to accurately characterize the main features of fungal composition related to sampling method. Alpha diversity indices did not show significant differences in fungal richness or distribution of taxa among samples (Supplementary Figure S8). Bray–Curtis diversity metrics was significantly different in back skin but not in elbow skin. Heat map representation of fungal abundances (the cut-off abundance was set at 1%) showed the strongest association with psoriatic back skin of the genus *Rhodotorula* followed by the genus *Penicillium*, order Capnodiales and species *Malassezia restricta* (Figure 3A). The species *Malassezia sympodialis* seemed to be more associated with unaffected skin of psoriatic patients, whereas the species *Debaryomyces hansenii* and *Malassezia globosa* were more associated with healthy control skin. Psoriatic elbow skin was characterized by the presence of *Candida railenensis*, whereas unaffected elbow skin by the genus *Rhodotorula*, species *Malassezia slooffiae*, *Naganishia diffluens* and the genus *Aspergillus*. Healthy elbow skin was associated with the genus *Penicillium* and the order Malasseziales (Figure 3B). LEfSe analysis focused on disease status (psoriatic, unaffected or healthy control skin) revealed a discriminative association of the species *Malassezia restricta* and the genus *Aspergillus* with psoriatic back skin, whereas the species *Penicillium miczynskii*, *Malassezia slooffiae* and the order Hypocreales were significantly associated with healthy back skin (Figure 3C). For elbow skin, the only species associated with psoriatic skin was *Malassezia sympodialis* (Figure 3D).

### Correlation Between Bacteria and Fungi Shows a Specific Pattern Related to Skin Condition and Sampling Site

**FIGURE 4:**
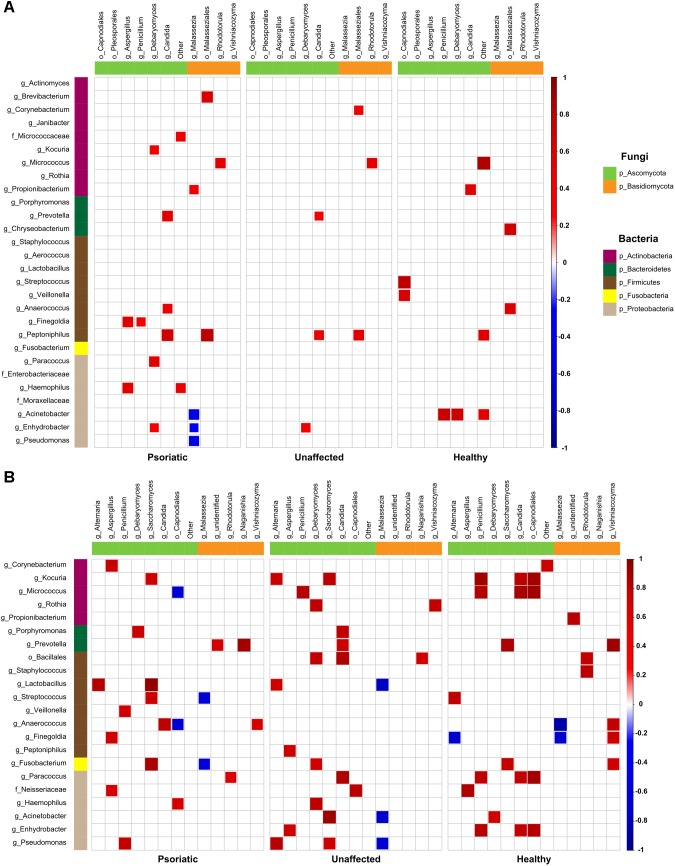
Bacteria–fungi correlation pattern in relation to psoriatic, unaffected or healthy control skin. Pearson correlation for the relative abundances of bacterial and fungal populations on the back **(A)** and elbow skin **(B)**. Correlations were calculated separately for each group of samples – psoriatic skin (left), unaffected skin (middle), and healthy skin (right). Positive correlations are represented by red squares; negative correlations are represented by blue squares, while insignificant correlations (*p* > 0.05) are blank.

Next, we investigated whether there is a correlation between fungal and bacterial constituents of the skin microbiome. We analyzed taxonomic correlations with body site and skin condition at genus and order levels. In psoriatic elbow skin, we found statistically significant positive correlation between occurrence of *Kocuria*, *Lactobacillus*, and *Streptococcus* with *Saccharomyces* (*r* = 0.73; *p* = 0.01 and *r* = 0.75; *p* = 0.01, respectively), which was not observed in healthy skin (*r* = 0.08; *p* = 0.84 and *r* = 0.03; *p* = 0.94, respectively). Interestingly, the genus *Micrococcus* was negatively correlated with *Capnodiales* in psoriatic skin (*r* = −0.69), in contrast to a positive correlation of these taxa in healthy control skin (*r* = 0.91). Other negative correlations were found to be specific for each skin condition and were not supported by positive correlation on the remaining sites (Figure 4A). On psoriatic back skin, we found negative correlation between the yeast *Malassezia* and three bacterial genera *Acinetobacter*, *Enhydrobacter*, and *Pseudomonas* (*r* = −0.59, *r* = −0.41 and *r* = −0.54). Similar to elbow skin, these negative correlations were not supported with positive associations, neither in unaffected nor in healthy skin (Figure 4B).

### Analysis of Intestinal Barrier Integrity Markers Shows Differences in I-FABP Levels

**FIGURE 5:**
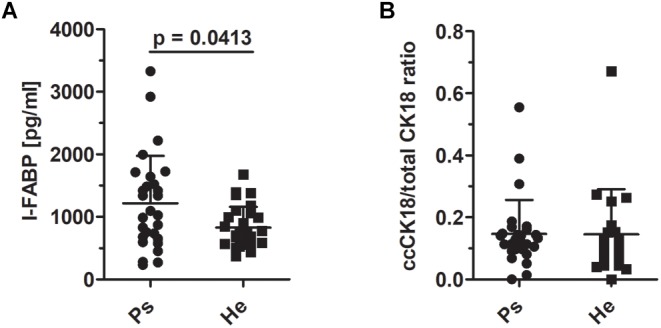
I-FABP levels and the ratio of cytokeratin 18 and caspase-cleaved cytokeratin 18 in sera. **(A)** The comparison of the levels of I-FABP (*p* = 0.0413, Mann–Whitney test) and **(B)** ccCK18/total CK18 ratios in psoriatic patients and healthy controls (*p* = 0.5034, Mann–Whitney test). Disease status: Ps, psoriatic patient; He, healthy control.

To test the hypothesis that enhanced epithelial disruption in the intestine is a present marker in patients with psoriasis, we measured intestinal fatty acid binding protein (I-FABP) and apoptotic index, the ratio of caspase-cleaved cytokeratin 18 fragment (ccCK 18) and total cytokeratin 18 (CK18) in the sera. The levels of I-FABP were significantly increased in patients with psoriasis compared to healthy controls (*p* = 0.0413) (Figure 5A). Nevertheless, we did not find any significant differences in the ration of ccCK 18/CK18 between psoriatic patients and healthy controls (*p* = 0.5034) (Figure 5B).

## Discussion

Several studies previously described differences in microbiota composition in psoriatic patients using dissimilar approaches (Tett et al., 2017; Yan et al., 2017). Here, we report a comprehensive analysis comparing all previously described methodological approaches in one data set. Moreover, for the first time, we include correlations between bacteria and fungi in psoriatic skin samples compared to healthy controls.

It is generally accepted that sequencing of various regions achieves different results when applied to the same samples (Hamady and Knight, 2009). Numerous combinations of primer pairs have been previously tested to select the most appropriate one for skin microbiome survey, but standardized methodology is still lacking (Meisel et al., 2016). Apart from appropriate but costly whole genome shotgun sequencing, primers for V1V3 and V3V4 hypervariable regions were described to sufficiently cover the skin bacterial diversity (Grice and Segre, 2011; Human Microbiome Project Consortium [HMPC], 2012a; Meisel et al., 2016; Castelino et al., 2017). In studies of the microbiome in psoriasis, both V1V3 and V3V4 sets of primers and nearly full length 16S rRNA have been used (Gao et al., 2008; Fahlen et al., 2012; Alekseyenko et al., 2013). Nevertheless, none of the published studies compared the suitability of V1V2 and V3V4 regions for characterizing skin microbial communities in psoriatic patients and healthy controls. For instance, Meisel et al., 2016 showed that using primers amplifying the V4 variable region resulted in a severe underrepresentation of several taxa, especially the genus *Propionibacterium* (Meisel et al., 2016). Our data indicate that this bias is not present when the V3V4 region is sequenced. Moreover, Meisel et al. (2016) were able to classify the majority of *Staphylococcus* using the V1V3 region, but not the V4 region. This is consistent with our finding that the V3V4 region was able to better capture genus *Staphylococcus* but primers for V1V2 region were better in classifying them to the species. However, our data show a marked absence of *Planococcaceae* when using the V3V4 region. In summary, we confirmed that the selection of primers used for studying the skin microbiota has an important impact on the resulting taxonomic coverage and thus for the further interpretation of the data. However, sequencing the V3V4 region resulted in a higher alpha-diversity overall.

Up to now, no common microbiota pattern in psoriatic patients has been identified across all the published work. This could be due to methodological differences, e.g., different sampling methods and sequencing of different variable regions, or due to a high degree of interindividual variation, specific niches of different sampling sites or low abundance of discriminatory taxa (Tett et al., 2017; Yan et al., 2017). For this reason, we sampled two different body sites by three different sampling techniques to be able to assess all of the above described aspects in one comprehensive study. However, not all participants were willing to provide biopsy sample, therefore we disposed of biopsies only from 10 patients. In contrast to other studies that compared various body sites in one study, we focused only on two well defined body sites – the elbow and the back, which are common sites frequently affected by psoriasis (Gao et al., 2008; Fahlen et al., 2012). One similar recent paper compared the elbow and the retroauricular crease as representative sites of psoriasis using shotgun sequencing, but unlike the present study focused only on isolates from swabs (Tett et al., 2017). Similarly as Tett et al. (2017) we tried to reduce the variability that arise from intraindividual and interindividual differences in microbiota composition by using control samples from unaffected skin from the same sampled patient. To deal with intra- and interindividual variation, we included not only samples of both psoriatic and contralateral unaffected skin from the same patient, but also samples from healthy controls.

Several host factors, including gender, age, place of residence, living with animals, hygiene habits, occupation, and ethnicity influence the composition of the skin microbiome (Fierer et al., 2008; Grice et al., 2009; Ying et al., 2015). Though, it seems that the effect of the psoriasis presence is much stronger than the effect of gender, since we have not found any significant changes in alpha diversity in psoriatic patients (Supplementary Figure S9). It has been also shown that different skin layers contain different bacterial communities (Grice et al., 2008; Kong et al., 2017). Nevertheless, the majority of previously published studies of the microbiome in psoriasis used only swabs as a single sampling technique (Tett et al., 2017; Yan et al., 2017). Here we compare swabs, scrapings, and biopsies to avoid potential sampling biases. Although due to ethical issues, we were not able to collect the biopsies from most of our tested individuals. In line with data on healthy skin (Grice et al., 2008), we found that the alpha diversity and the presence of main skin bacterial taxa in all sampling techniques are comparable. Moreover, we show that this holds irrespective of disease status. However, we observed higher richness and evenness in psoriatic skin on the elbow compared to psoriatic back skin, which is consistent with previous research and possibly reflects environmental differences between these two microhabitats (Tett et al., 2017). Comparing the microbial heterogeneity, we have not detected significant beta diversity differences between psoriatic lesions and unaffected psoriatic skin nor on the back neither on the elbow. Additionally, our analysis revealed several minor differences depending on the sampling approach. The relative abundance of *Streptococcus* in swabs, scrapings and biopsies was higher in psoriatic lesions compared to controls regardless of the sampling site, which is consistent with previous findings (Fahlen et al., 2012; Alekseyenko et al., 2013). In contrast, the abundance of *Propionibacterium* was lower in psoriatic lesions and unaffected psoriatic skin compared to healthy skin only on the elbow but not on the back. This highlights the importance of site-specific niches, e.g., different microbiota composition at oily and dry skin sites (Grice et al., 2008; Fahlen et al., 2012; Belkaid and Segre, 2014). In agreement with the findings of Fahlen et al. (2012), we described lower abundances of *Staphylococcus* in psoriatic skin biopsies (Fahlen et al., 2012). On the other hand, *Actinobacteria* and *Propionibacterium* were lower in biopsies of healthy skin.

As might be expected, we found that many of the identified metabolic pathways are common to all samples and can be assigned to “core” pathways. Most metabolic pathways of elbow skin microbiota uncovered in our metagenomic study overlap with those previously described (Tett et al., 2017). We did not observe any niche-specific variations in the distribution of the most abundant KEGG pathways in the elbow and back samples. Interestingly, we identified the ethylbenzene degradation pathway as the only discriminative pathway common for unaffected psoriatic skin on both sites. This could be connected to the increase in abundance of *Pseudomonas*, which are known to utilize ethylbenzene as a source of energy (Utkin et al., 1991). We also found a significantly lower abundance of Notch signaling pathway in psoriatic skin compared to healthy skin. This is in concordance with previously reported data about Notch signaling, showing that alterations in this pathway, together with aberrant expression of keratin 10 and keratin 14, is associated with abnormal keratinocyte differentiation leading to unorganized suprabasal epidermal strata (Thelu et al., 2002; Ota et al., 2014).

Studies concerning the mycobiome composition in psoriasis are still scarce and no prior studies assessed the impact of different sampling methods on the recovered mycobiome composition. We observed comparable alpha diversity and microbial patterns in the skin mycobiome regardless of the sampling technique used. In comparison to study by Findley et al. (2013), we detected higher number of genera. This dissimilarity could be caused by different strategy in clustering procedure. Our study substantiates previous findings that *Malassezia* is the dominant fungal genus occurring on the human skin and that psoriatic lesions display greater fungal diversity than healthy skin (Findley et al., 2013). Moreover, we detected that psoriatic lesions on the back are predominated by *M. restricta* as previously described by Paulino et al. (2006), followed by *M. globosa* and *M. sympodialis*, and we found no consistent dichotomous differences between the tested groups. In agreement with Takemoto et al. (2015), we observed that the ratio of *M. globosa* to *M. restricta* is lower in psoriatic lesions on the back compared to healthy skin (Takemoto et al., 2015). The same pattern is evident on the elbow, where the ratio was lower in psoriasis patients, both in lesions and in unaffected skin. Psoriatic lesions on the elbow were further characterized by a significantly higher abundance of *M. sympodialis* compared to healthy skin. However, this was not true for back skin, which emphasize the need to keep in mind the differences in mycobiome composition in different skin niches, for example when comparing oily and dry sites. Moreover, not only skin niches but also ethnicity probably plays an important role in *Malassezia* presence in the psoriatic lesions as reviewed by Prohic et al. (2016). For example, in a study cohort of Polish patients, *M. sympodialis* was the predominant species, whereas in the psoriatic skin of Canadian patients *M. globosa* was the most common (Gupta et al., 2001; Jagielski et al., 2014). In contrast with these studies, Japanese patients mainly harbored *M. restricta* (Amaya et al., 2007). The mechanism by which *Malassezia* could contribute to the pathogenesis of psoriasis is not yet fully described, but it is known that *M. sympodialis* can enhance the production of pro-inflammatory cytokines IL-1 (interleukin 1), TNF-α (tumor necrosis factor alpha), IL-8 (interleukin 8), and IL-6 (interleukin 6) in keratinocytes (Watanabe et al., 2001). Moreover, *M. sympodialis* can induce uncontrolled pro-inflammatory maturation of dendritic cells and activation of mast cells, which release leukotrienes, which are increased in patients with psoriasis (Fauler et al., 1992; Buentke et al., 2002; Selander et al., 2009).

Our study is the first to conduct a simultaneous analysis of both bacterial and fungal microbiota to clarify the disease-specific inter-kingdom differences between patients with psoriasis and healthy controls. Consistently with a study on healthy skin (Findley et al., 2013), we observed more frequent bacteria–fungi equilibrium on the elbow than on the back, which reflects the different microenvironments. This suggests a greater importance of such equilibrium in mutual relationships or competition in dry and more exposed skin compared to sebaceous skin sites. Recently, an important interaction between *Lactobacillus* and *Streptococcus* has been described (Saroj et al., 2016). Here, we notice its importance in psoriatic patients together with concurrent abundance of *Saccharomyces.* Further studies are needed to achieve a more thorough understanding of potential inter-kingdom interactions in the skin microbiome with emphasis on their role in the pathogenesis of psoriasis.

There is growing evidence which emphasize the importance of the gut-skin axis in the pathogenesis of psoriasis (Mattozzi et al., 2012; Yan et al., 2017). Fox example, a study showing that medical treatment of the so-called small intestinal bacterial overgrowth syndrome could mitigate psoriasis (Drago et al., 2018), suggests an undeniable importance of microbiome in psoriasis pathogenesis. Moreover, recent study by Tan et al. (2018) described decreased abundance of *Akkermansia muciniphila*, an important producer of short fatty acid binding protein and mucin degrading bacteria, in the gut of psoriatic patients (Tan et al., 2018). We found that levels of I-FABP, a marker of cell epithelial damage, were increased in psoriatic patients in comparison to healthy controls which is in agreement with study by Sikora et al. (2018). Since the disruption of intestinal barrier seems to play a role in disease pathogenesis we searched for another marker of enterocyte damage – the ratio of ccCK18/CK18, but we did not find any significant differences between psoriatic patients and healthy controls. Nevertheless, more studies are needed to describe other markers of intestinal barrier disruption and their effects on psoriasis pathogenesis with the final goal to develop a new preventive or treatment options for psoriatic patients.

## Conclusion

In conclusion, the data reported here extend our understanding of microbiota composition in psoriatic patients. We provide a unique insight into disease-specific inter-kingdom network alterations and highlight the importance of viewing bacteria and fungi as important interconnected players in disease pathogenesis. A deeper understanding of the complex microbial ecosystem is needed to be able to modulate the equilibrium therapeutically by using probiotics, antimicrobials and even topical microbiota transplantation (Langan et al., 2018; Myles et al., 2018).

## Author Contributions

HT-H, JH, KJ, OK, and ZJ conceived and designed the research. KJ, FR, and PB examined the patients and collected the samples. ZS performed the experiments. ZS, MKo, MKv, KK, YP, AU, and ZJ analyzed and interpreted the data. ZS, KK, OK, and ZJ wrote the manuscript. All authors revised and approved the final version of the manuscript.

## Conflict of Interest Statement

The authors declare that the research was conducted in the absence of any commercial or financial relationships that could be construed as a potential conflict of interest. The reviewer JH declared a shared affiliation, with no collaboration, with several of the authors, KJ, FR, JH, and PB, to the handling editor at the time of review.

## Abbreviations

Bio: biopsies
He: healthy
Ps: psoriatic
Scr: scrapings
Swa: swabs
Un: unaffected.
