# Supplementary material for: Dysbiosis of Skin Microbiota in Psoriatic Patients: Co-occurrence of Fungal and Bacterial Communities
Source: Front Microbiol. 2019 Mar 21;10:438. doi: 10.3389/fmicb.2019.00438 (PMC6437110; doi:10.3389/fmicb.2019.00438)
Supplement: Supplementary file 1 [file Data_Sheet_1.pdf]

Supplementary Material

Dysbiosis of skin microbiota in psoriatic patients: co-occurrence of fungal and bacterial communities

Stehlikova Z. [1], Kostovcik M. [1,2], Kostovcikova K.[1], Kverka M.[1,3], Juzlova K.[4], Rob F.[4], Hercogova J.[4], Bohac P.[4], Pinto Y.[5], Uzan A.[5], Koren O.[5], Tlaskalova-Hogenova H.[1] and Jiraskova Zakostelska Z. [1*]

[1]Institute of Microbiology, Czech Academy of Sciences, Prague, Czech Republic.

[2]BIOCEV, Institute of Microbiology, Academy of Sciences of the Czech Republic, Vestec, Czech Republic

[3]Institute of Experimental Medicine of the Czech Academy of Sciences, v.v.i., Prague, Czech Republic

[4]Bulovka Hospital, 2nd Faculty of Medicine, Dept. of Dermatology, Charles University in Prague, Prague, Czech Republic

[5]The Azrieli Faculty of Medicine, Bar-Ilan University, Safed, Israel

***Correspondence:**

Dr. Zuzana Jiraskova Zakostelska

zakostelska@biomed.cas.cz

1. SUPPLEMENTARY TABLES AND FIGURES

1.1. Tables related to Material and methods

Table S1. Characteristics of patients and controls in the study.

		**Range (average ± SD)**			
		**Age**	**BMI**	**PASI**	**PGA**
**Psoriasis**	**female** (n = 6)	24 – 49 (37,2 ± 7,8)	22 – 37 (29,1 ± 5,3)	0,5 – 32 (10,5 ± 10,3)	1 – 4 (2,5 ± 0,96)
	**male** (n = 28)	23 – 70 (46,4 ± 11,9)	22 – 49 (29,7 ± 5,9)	0 – 20 (5,1 ± 5,6)	0 – 4 (1,9 ± 1)
Total	n = 34	23 – 70 (44,8 ± 11,9)	22 – 49 (29,6 ± 5,8)	0 – 32 (6,1 ± 6,9)	0 – 4 (2 ± 1)
**Controls**	**female** (n = 14)	27 – 72 (41,7 ± 14,8)	–	–	–
	**male** (n = 13)	22 – 69 (42,6 ± 14,6)	–	–	–
Total	n = 27	22 – 72 (44 ± 13,3)	–	–	–

Table S2. Medical and sampling history of psoriatic patients.

	Treatment				Sampling		
**Patient ID**	**Biological**	**Systemic**	**None**	**Other**	**Swab**	**Scraping**	**Biopsy**
P1	x	–	–	–	x	x	–
P2	x	–	–	–	x	x	x
P7	–	x	–	–	x	x	–
P8	–	x	–	–	x	x	–
P9	x	–	–	–	x	x	x
P10	–	–	–	x	x	x	–
P11	x	–	–	–	x	x	x
P12	–	x	–	–	x	x	x
P13	–	–	–	x	x	x	x
P14	–	–	–	x	x	x	x
P15	–	–	x	–	x	x	x
P16	x	–	–	–	x	x	x
P17	x	–	–	–	x	x	x
P18	x	–	–	–	x	x	–
P19	x	–	–	–	x	x	–
P22	–	x	–	–	x	x	–
P23	x	–	–	–	x	x	–
P26	–	x	–	–	x	x	–
P31	–	–	–	x	x	x	–
P32	x	–	–	–	x	x	–
P33	–	–	–	x	x	x	–
P35	x	–	–	–	x	x	–
P36	x	–	–	–	x	x	–
P37	x	–	–	–	x	x	–
P41	x	–	–	–	x	x	–
P44	–	–	–	x	x	x	–
P46	–	–	–	x	x	x	–
P50	–	–	x	–	x	x	–
P51	–	–	x	–	x	x	–
P52	–	–	x	–	x	x	–
P54	–	–	x	–	x	x	–
P55	–	–	x	–	x	x	–
P56	–	–	x	–	x	x	–
P57	–	–	x	–	x	x	–
**Total patients**	**14**	**5**	**8**	**7**	**34**	**34**	**9**

Table S3. Sampling of psoriatic, unaffected psoriatic and healthy skin.

		Localization		
	**Psoriatic**	**Back**	**Elbow**	**Total count**
**Psoriasis**	swabs	25	9	34
	scraping	25	9	34
	biopsy	10	0	10
	**Unaffected**			
	swabs	25	9	34
	scraping	25	9	34
	biopsy	9	0	9
Number of patients		**25**	**9**	**34**
**Controls**	**Healthy**			
	swabs	17	8	25
	scraping	15	8	23
	biopsy	7	1	8
Number of controls		**19**	**8**	**27**

1.2 Figures and tables related to Results

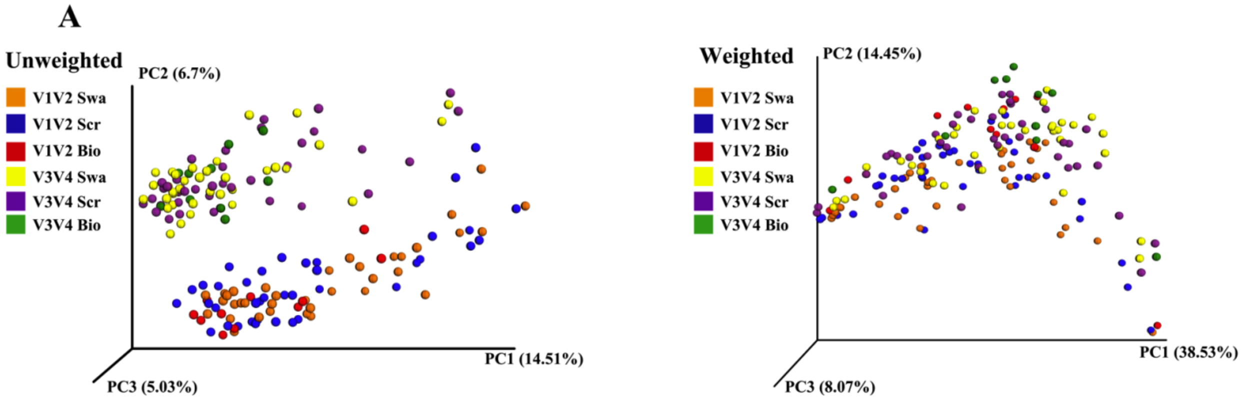

PCoA Unweighted	p-value	PCoA Weighted	p-value
V1V2 Swa vs. V3V4 Swa	0.001	V1V2 Swa vs. V3V4 Swa	0.001
V1V2 Scr vs. V3V4 Scr	0.001	V1V2 Scr vs. V3V4 Scr	0.001
V1V2 Bio vs. V3V4 Bio	0.001	V1V2 Bio vs. V3V4 Bio	0.389

Figure S1. (related to Figure 1) **Differences in microbiota composition due to sequencing of V1V2 and V3V4 16S rRNA regions. (A)** PCoA of Weighted and Unweighted UniFrac distances (beta diversity) between the respective regions and type of sampling (swabs, scraping, biopsy). Statistical significance was confirmed using PERMANOVA. *$p < 0.05$; **$p < 0.01$, and ***$p < 0.001$. See the table above. Type of sampling: Swa, swab; Scr, scraping; Bio, biopsy .

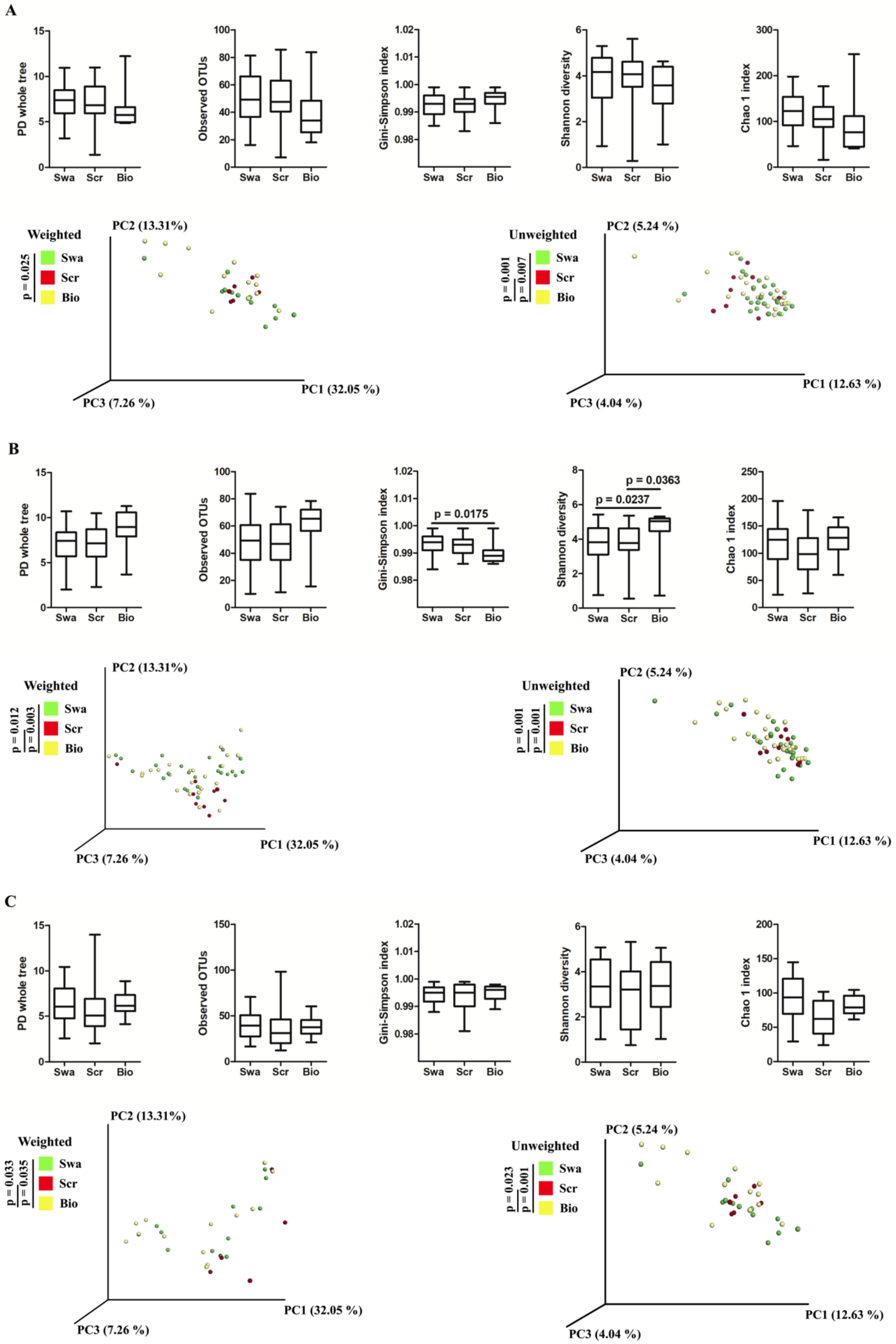
A
PD whole tree
Observed OTUs
Gini-Simpson index
Shannon diversity
Chao 1 index
Swa
Scr
Bio
Weighted
p = 0.025
PC2 (13.31%)
PC1 (32.05 %)
PC3 (7.26 %)
Unweighted
p = 0.001
p = 0.007
PC2 (5.24 %)
PC1 (12.63 %)
PC3 (4.04 %)
B
p = 0.0175
p = 0.0237
p = 0.0363
p = 0.012
p = 0.003
p = 0.001
p = 0.001
C
p = 0.033
p = 0.035
p = 0.023
p = 0.001

Figure S2. (related to Table 1) **Different sampling approaches result in similar bacterial diversity but differ in genera abundance in back. (A)** Alpha diversity (PD whole tree, Observed OTU´s, Gini-Simpson, Shannon and Chao1 index) and beta diversity indices (weighted and unweighted PCoA analysis) of psoriatic skin showing microbial richness and distribution in relation to swab, scraping and biopsy sampling approach. **(B)** Alpha and beta diversity indices of unaffected psoriatic skin showing microbial richness and distribution in relation to swab, scraping and biopsy sampling approach. **(C)** Alpha and beta diversity indices of healthy skin showing microbial richness and distribution in relation to swab, scraping and biopsy sampling approach. Statistical significance was confirmed using Kruskal–Wallis test with Dunn's multiple comparison test and PERMANOVA. Sampling approaches: Swa, swabs; Scr, scrapings; Bio, biopsies. Significant differences are noted by $^{*}p < 0.05$, $^{**}p < 0.01$, and $^{***}p < 0.001$.

A

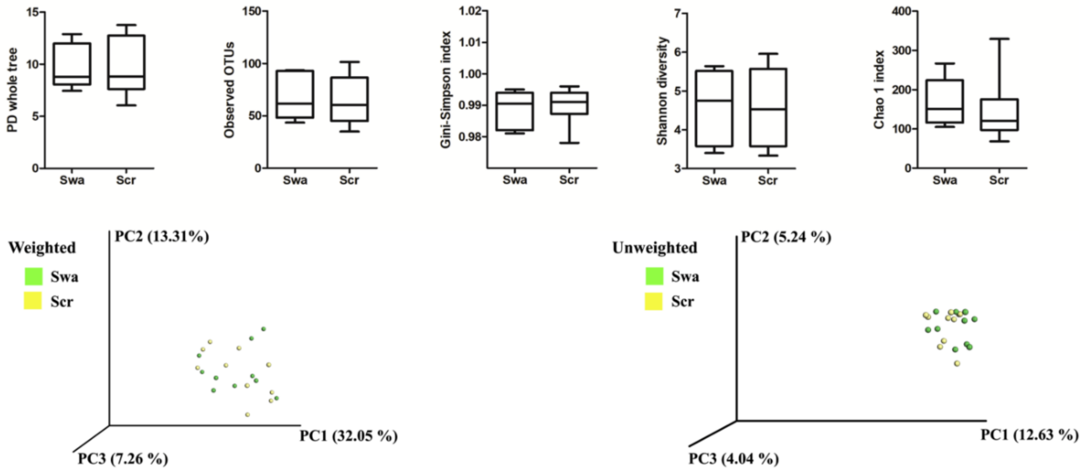
PD whole tree
Observed OTUs
Gini-Simpson index
Shannon diversity
Chao 1 index
Swa
Scr
Weighted
Unweighted
PC1 (32.05 %)
PC2 (13.31%)
PC3 (7.26 %)
PC1 (12.63 %)
PC2 (5.24 %)
PC3 (4.04 %)

B

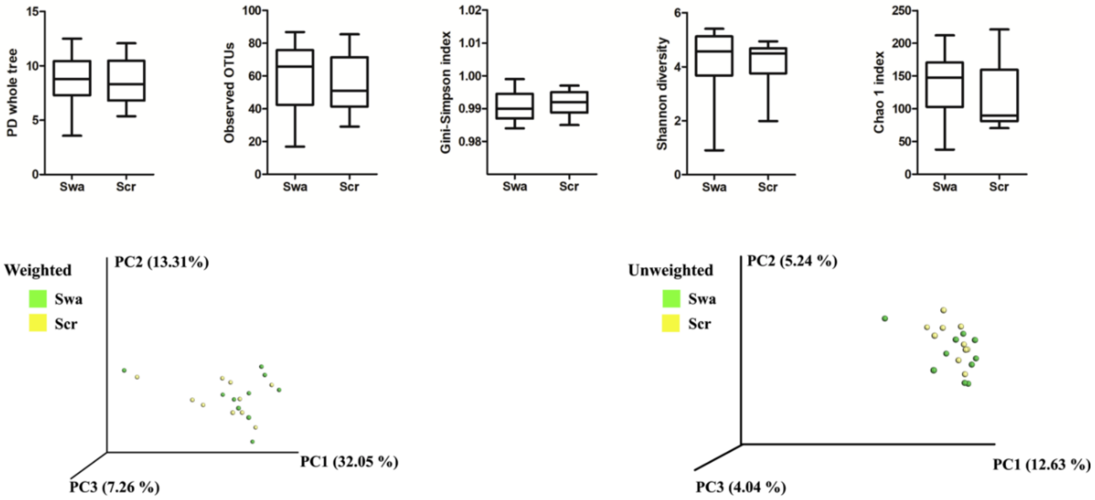
PD whole tree
Observed OTUs
Gini-Simpson index
Shannon diversity
Chao 1 index
Swa
Scr
Weighted
Unweighted
PC1 (32.05 %)
PC2 (13.31%)
PC3 (7.26 %)
PC1 (12.63 %)
PC2 (5.24 %)
PC3 (4.04 %)

C

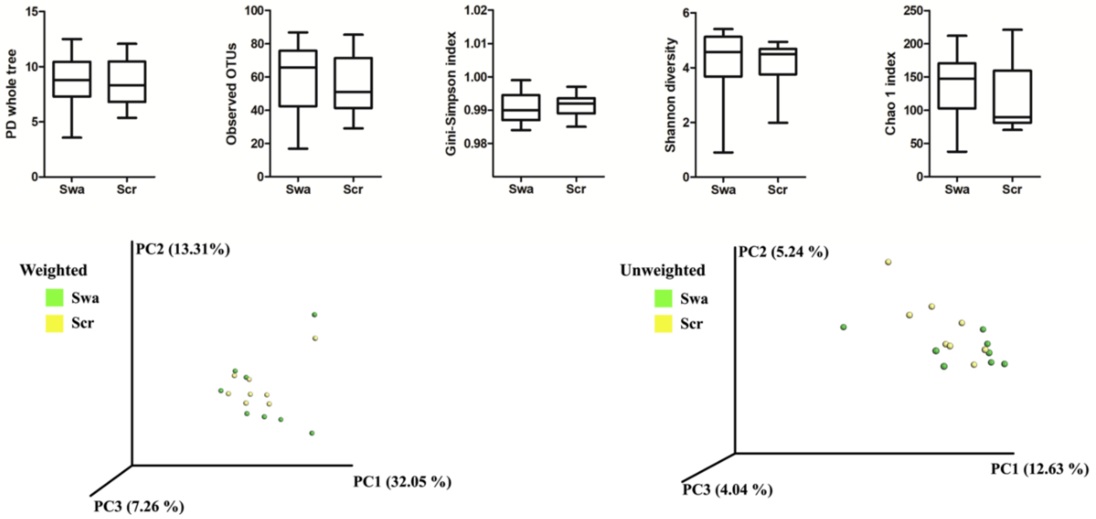
PD whole tree
Observed OTUs
Gini-Simpson index
Shannon diversity
Chao 1 index
Swa
Scr
Weighted
Unweighted
PC1 (32.05 %)
PC2 (13.31%)
PC3 (7.26 %)
PC1 (12.63 %)
PC2 (5.24 %)
PC3 (4.04 %)

Figure S3. (related to Table 1) **Different sampling approaches result in similar microbial diversity but differ in genera abundance in elbow. (A)** Alpha diversity (PD whole tree, Observed OTU´s, Gini-Simpson, Shannon and Chao1 index) and beta diversity indices (weighted and unweighted PCoA analysis) of psoriatic skin showing microbial richness and distribution in relation to swab and scraping sampling approach. **(B)** Alpha and beta diversity indices of unaffected psoriatic skin showing microbial richness and distribution in relation to swab and scraping sampling approach. **(C)** Alpha and beta diversity indices of healthy skin showing microbial richness and distribution in relation to swab and scraping sampling approach. Statistical significance was confirmed using Mann-Whitney test and PERMANOVA. Sampling approaches: Swa, swabs; Scr, scrapings. Significant differences are noted by $*p < 0.05$, $**p < 0.01$, and $***p < 0.001$.

Table S4. (related to Table 1) **Differences in bacterial distribution related to sampling approach.** (Swa, swabs; Scr, scrapings; Bio, biopsies).

			Streptococcus	*Propionibacterium*	Actinobacteria	*Staphylococcus*
Back	Psoriatic	Swa	3.1	20.0	52.8	20.3
		Scr	3.5	30.6	58.9	14.6
		Bio	0.5	37.0	55.4	12.1
	Unaffected	Swa	2.6	18.4	46.4	26.6
		Scr	5.3	20.4	41.9	31.7
		Bio	0.7	25.2	44.8	4.6
	Healthy	Swa	1.8	28.3	43.3	26.9
		Scr	0.7	51.3	61.4	19.5
		Bio	0.4	8.0	15.2	55.4
Elbow	Psoriatic	Swa	4.8	8.3	29.3	18.7
		Scr	6.7	11.3	27.9	13.7
	Unaffected	Swa	2.5	15.8	39.5	20.4
		Scr	3.2	13.8	33.3	26.5
	Healthy	Swa	2.7	22.9	53.5	20.5
		Scr	3.8	23.5	49.4	25.8

Table S5. (related to Table 1) **The main discriminative bacterial features related to sampling approaches of V1V2 region.**

Representative bacterial biomarkers related to sampling approaches			
Sample site		Sample type	
		Swabs	Scrapings
Back	Psoriatic	-	*Bacillus*<br>Moraxellaceae<br>*Comamonas*<br>*Burkholderia*<br>*Veillonella*<br>Pseudomonadales<br>Gammaproteobacteria<br>Burkholderiales
	Unaffected	Lachnospiraceae	*Neisseria*<br>*Granullicatella*<br>Gammaproteobacteria<br>Oxalobacteraceae<br>Burkholderiales<br>Comamonadaceae
	Healthy	-	-

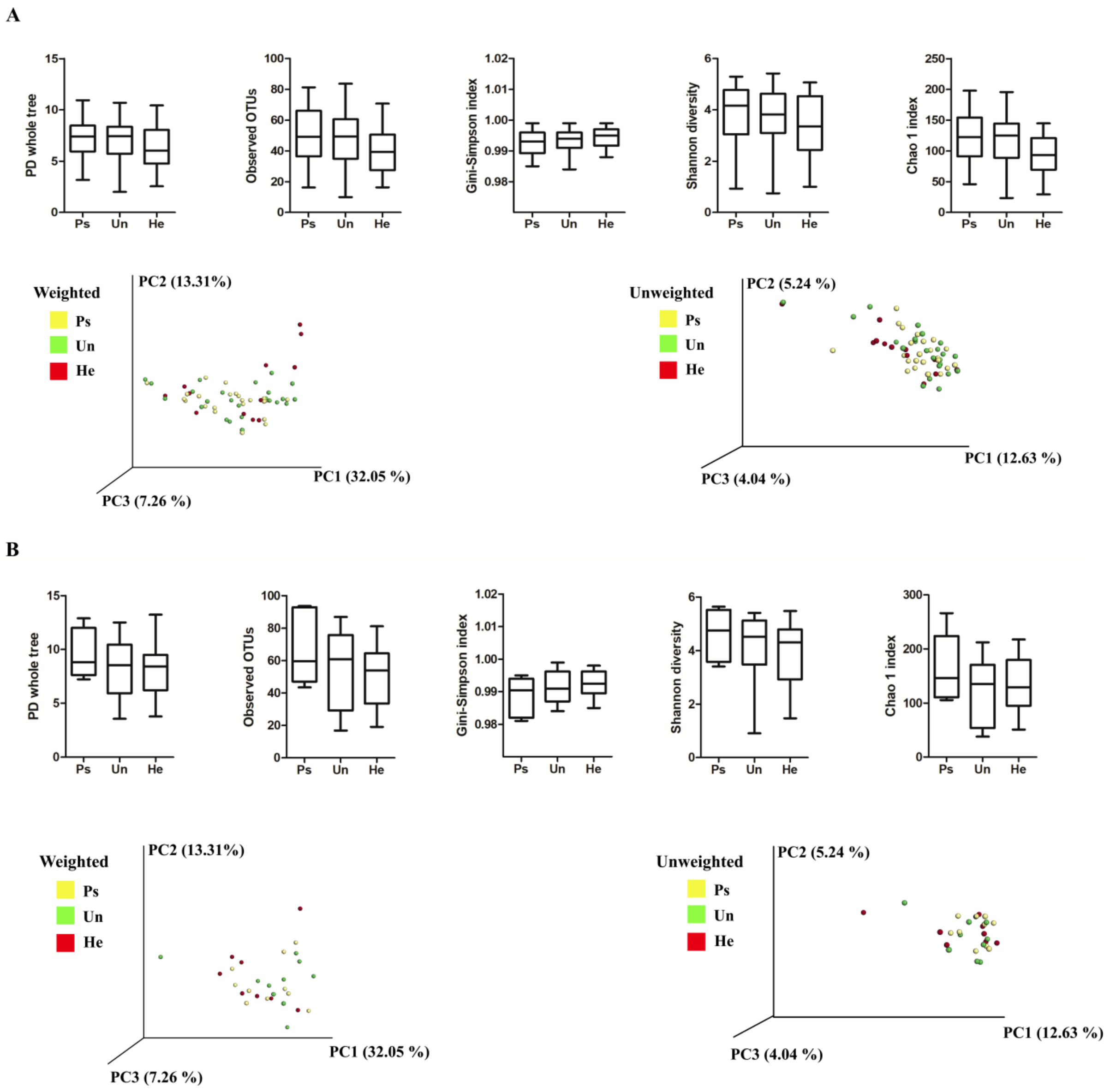

Figure S4. (related to Figure 2) **Analysis of bacterial communities isolated from swabs using the V3V4 region of the 16S rRNA gene. (A)** Alpha diversity (PD whole tree, Observed OTU´s, Gini-Simpson, Shannon and Chao1 index) and beta diversity indices (weighted and unweighted PCoA analysis) of psoriatic, unaffected psoriatic and healthy control skin in back samples. **(B)** Alpha diversity (PD whole tree, Observed OTU´s, Gini-Simpson, Shannon and Chao1 index) and beta diversity indices (weighted and unweighted PCoA analysis) of psoriatic, unaffected psoriatic and healthy control skin in elbow samples. Disease status: Ps, psoriatic; Un, unaffected; He, healthy skin. Statistical significance was confirmed using Kruskal–Wallis test with Dunn's multiple comparison test and PERMANOVA. Significant differences are noted by $*p < 0.05$, $**p < 0.01$, and $***p < 0.001$.

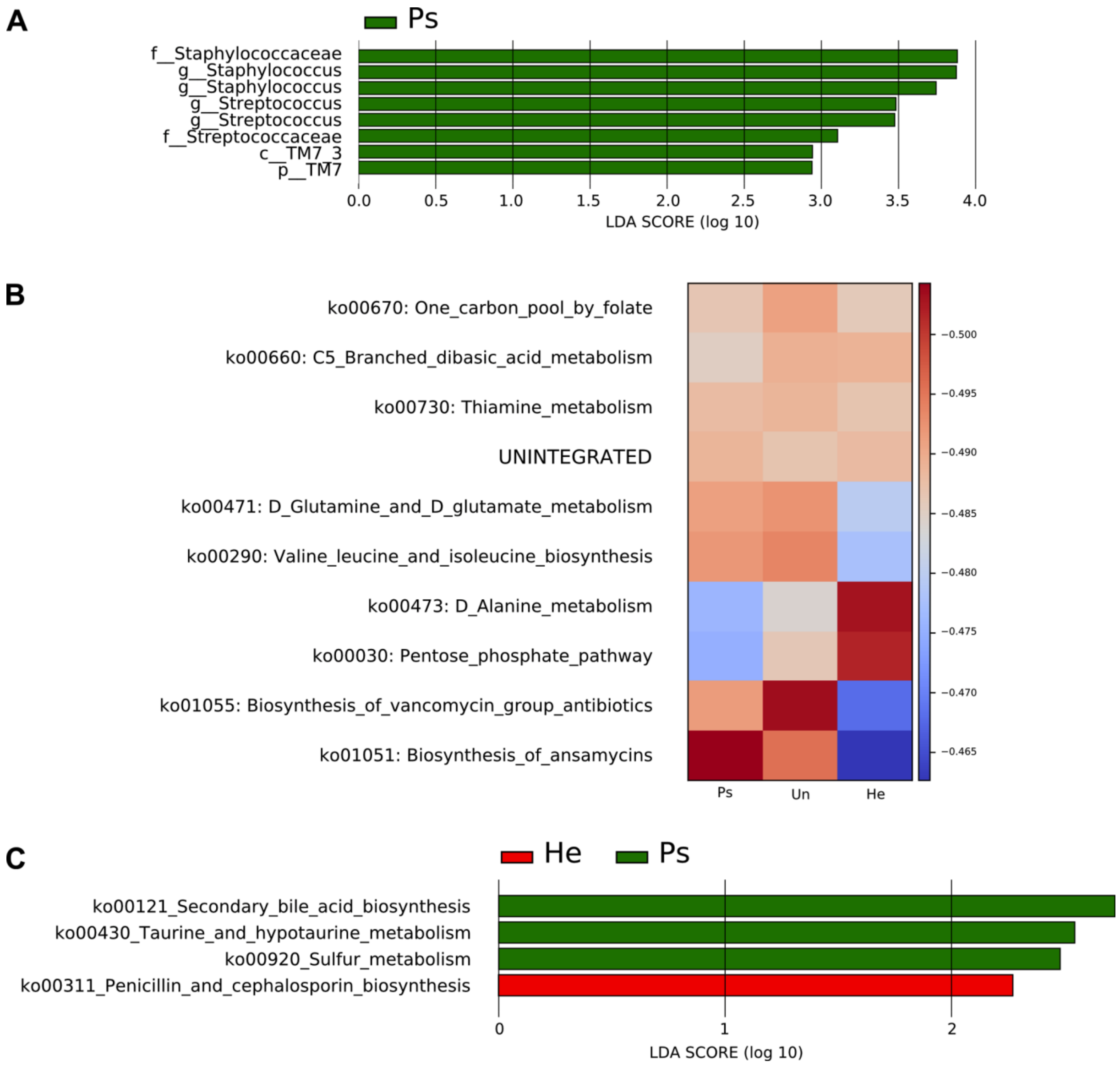

Figure S5. (related to Figure 2) **Analysis of bacterial communities isolated from swabs using the V1V2 region of the 16S rRNA gene. (A)** LEfSe analysis showed microbial patterns that were significantly differentially abundant in back samples. **(B)** Predicted functional profiles of metabolic pathways in back samples associated with psoriatic, unaffected or healthy control skin. Heat map include only KEGG pathways with abundance above 1% for each localization and disease status. The differences are shown by color code depending on the relative abundance of metabolic pathway. Red: High abundance; Blue: Low abundance. **(C)** LEfSe analysis displaying discriminative metabolic patterns in back samples. Disease status: Ps, psoriatic; Un, unaffected; He, healthy skin.

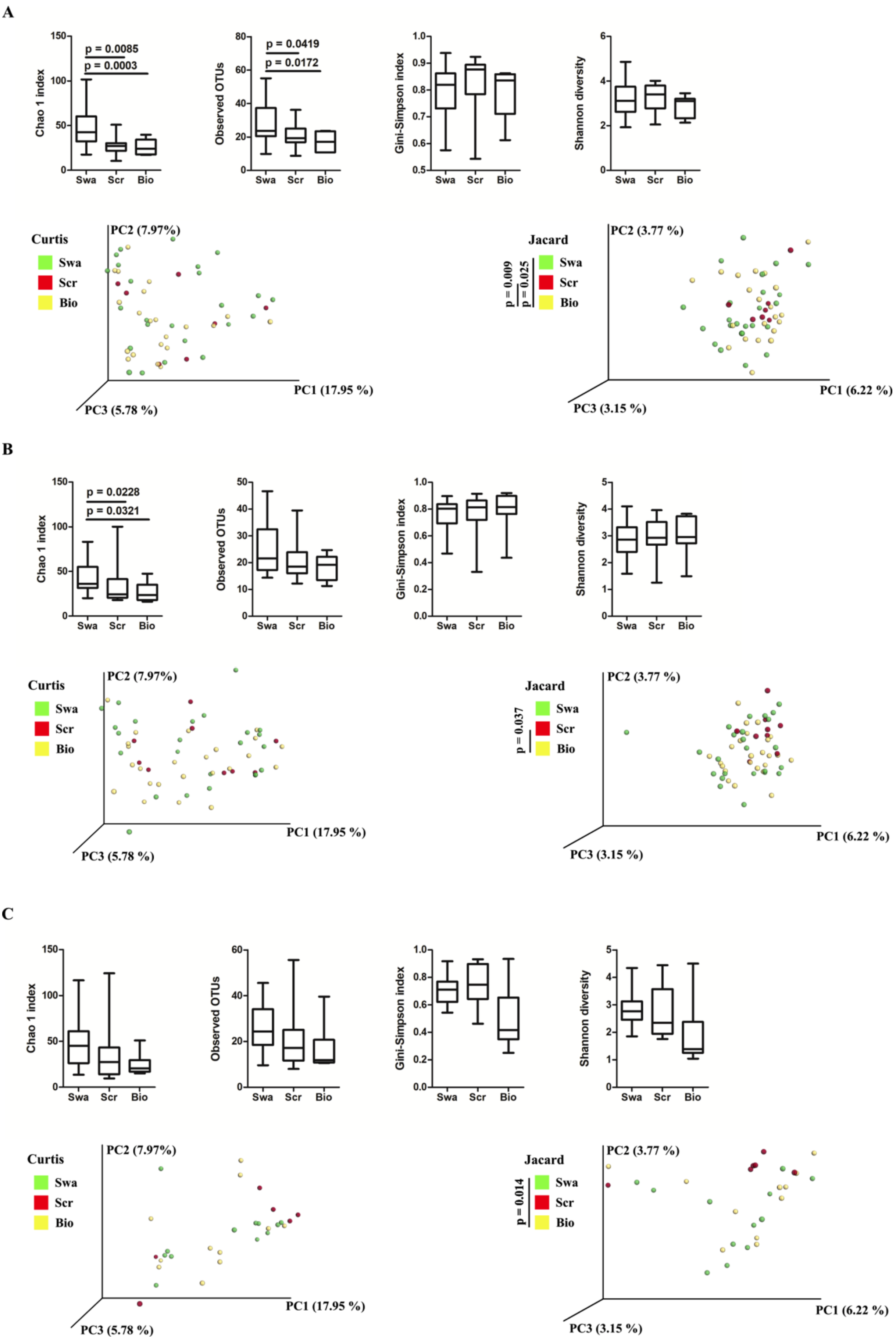
A
Chao 1 index
p = 0.0085
p = 0.0003
Observed OTUs
p = 0.0419
p = 0.0172
Gini-Simpson index
Shannon diversity
Swa Scr Bio
Curtis
Swa
Scr
Bio
PC2 (7.97%)
PC1 (17.95 %)
PC3 (5.78 %)
Jacard
p = 0.009
p = 0.025
PC2 (3.77 %)
PC1 (6.22 %)
PC3 (3.15 %)
B
p = 0.0228
p = 0.0321
p = 0.037
C
p = 0.014

Figure S6. (related to Table 2) **Different sampling approaches result in similar fungal diversity but differ in genera abundance in back. (A)** Alpha diversity (PD whole tree, Observed OTU´s, Gini-Simpson, Shannon and Chao1 index) and beta diversity indices (weighted and unweighted PCoA analysis) of psoriatic skin showing fungal richness and distribution in relation to swab, scraping and biopsy sampling approach. **(B)** Alpha and beta diversity indices of unaffected psoriatic skin showing fungal richness and distribution in relation to swab, scraping and biopsy sampling approach. **(C)** Alpha and beta diversity indices of healthy skin showing fungal richness and distribution in relation to swab, scraping and biopsy sampling approach. Statistical significance was confirmed using Kruskal–Wallis test with Dunn's multiple comparison test and PERMANOVA. Sampling approaches: Swa, swabs; Scr, scrapings; Bio, biopsies. Significant differences are noted by $^{*}p < 0.05$, $^{**}p < 0.01$, and $^{***}p < 0.001$.

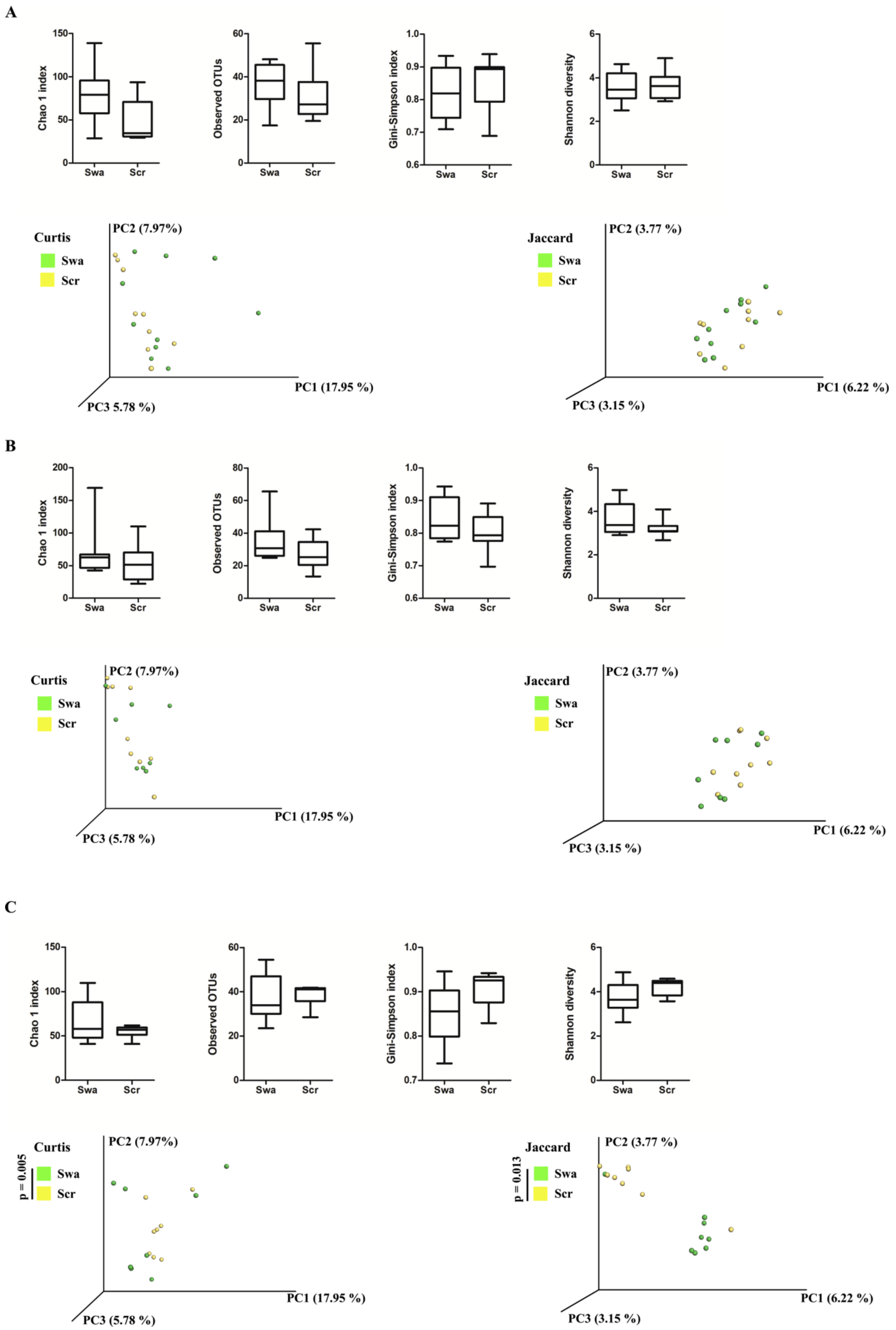
A
Chao 1 index
Observed OTUs
Gini-Simpson index
Shannon diversity
Swa
Scr
Curtis
PC2 (7.97%)
PC1 (17.95 %)
PC3 5.78 %)
Jaccard
PC2 (3.77 %)
PC1 (6.22 %)
PC3 (3.15 %)
B
Chao 1 index
Observed OTUs
Gini-Simpson index
Shannon diversity
Curtis
PC2 (7.97%)
PC1 (17.95 %)
PC3 (5.78 %)
Jaccard
PC2 (3.77 %)
PC1 (6.22 %)
PC3 (3.15 %)
C
Chao 1 index
Observed OTUs
Gini-Simpson index
Shannon diversity
Curtis
p = 0.005
PC2 (7.97%)
PC1 (17.95 %)
PC3 (5.78 %)
Jaccard
p = 0.013
PC2 (3.77 %)
PC1 (6.22 %)
PC3 (3.15 %)

Figure S7. (related to Table 2) **Different sampling approaches result in similar fungal diversity but differ in genera abundance in elbow. (A)** Alpha diversity (PD whole tree, Observed OTU´s, Gini-Simpson, Shannon and Chao1 index) and beta diversity indices (Bray-Curtis, Binary-Jaccard) of psoriatic skin showing fungal richness and distribution in relation to swab and scraping sampling approach. **(B)** Alpha and beta diversity indices of unaffected psoriatic skin showing fungal richness and distribution in relation to swab and scraping sampling approach. **(C)** Alpha and beta diversity indices of healthy skin showing fungal richness and distribution in relation to swab and scraping sampling approach. Statistical significance was confirmed using Mann-Whitney test and PERMANOVA. Sampling approaches: Swa, swabs; Scr, scrapings. Significant differences are noted by *p < 0.05, **p < 0.01, and ***p < 0.001.

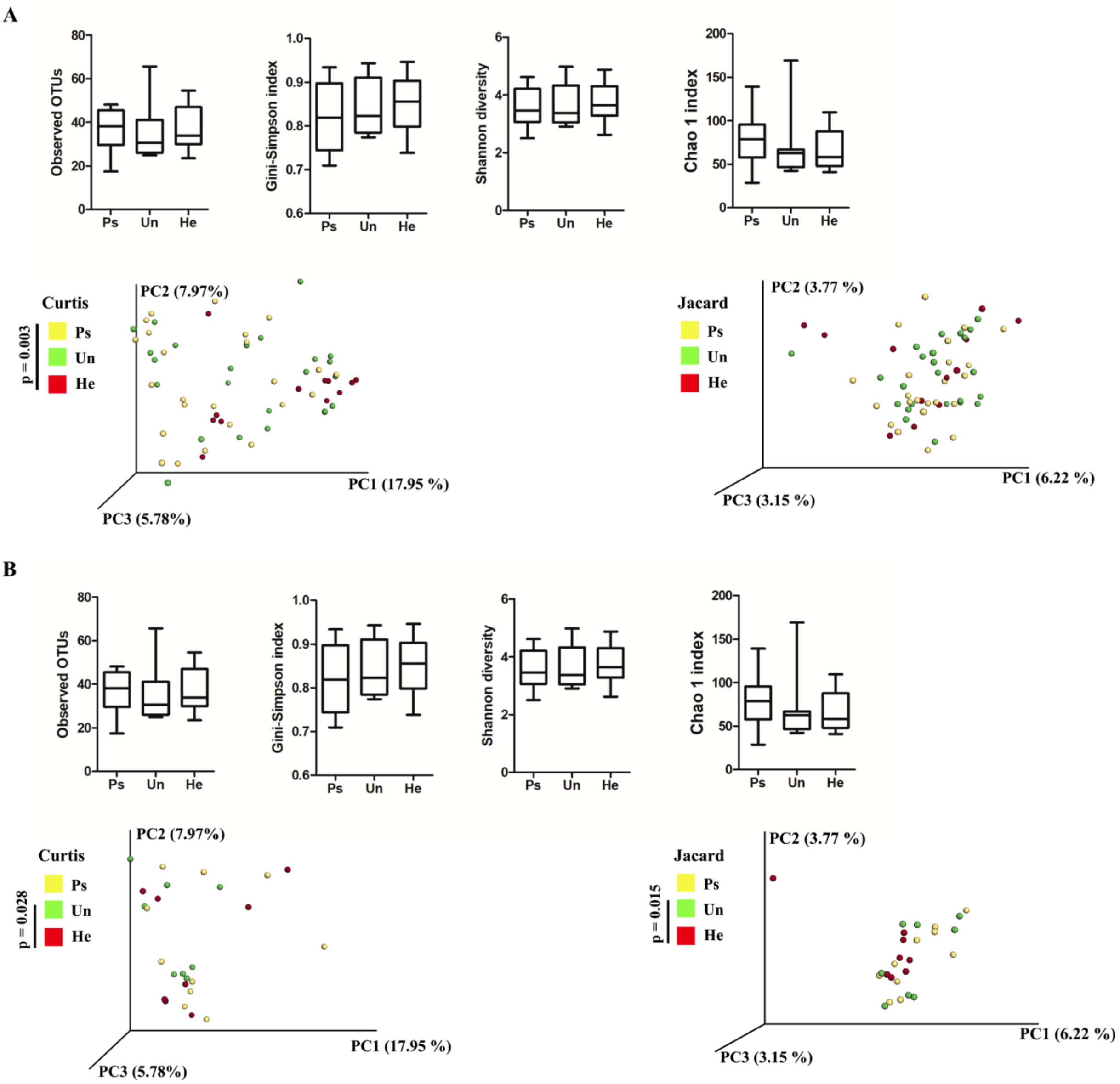

Figure S8. (related to Figure 3) **Analysis of fungal communities isolated from swabs. (A)** Alpha diversity (PD whole tree, Observed OTU´s, Gini-Simpson, Shannon and Chao1 index)

and beta diversity metrics (Bray-Curtis, Binary-Jaccard) of psoriatic, unaffected psoriatic and healthy control skin in back samples. **(B)** Alpha and beta diversity indices of psoriatic, unaffected psoriatic and healthy control skin in elbow samples. Disease status: Ps, psoriatic; Un, unaffected; He, healthy skin. Statistical significance was confirmed using Kruskal–Wallis test with Dunn's multiple comparison test and PERMANOVA. Significant differences are noted by *p < 0.05; **p < 0.01, and ***p < 0.001.

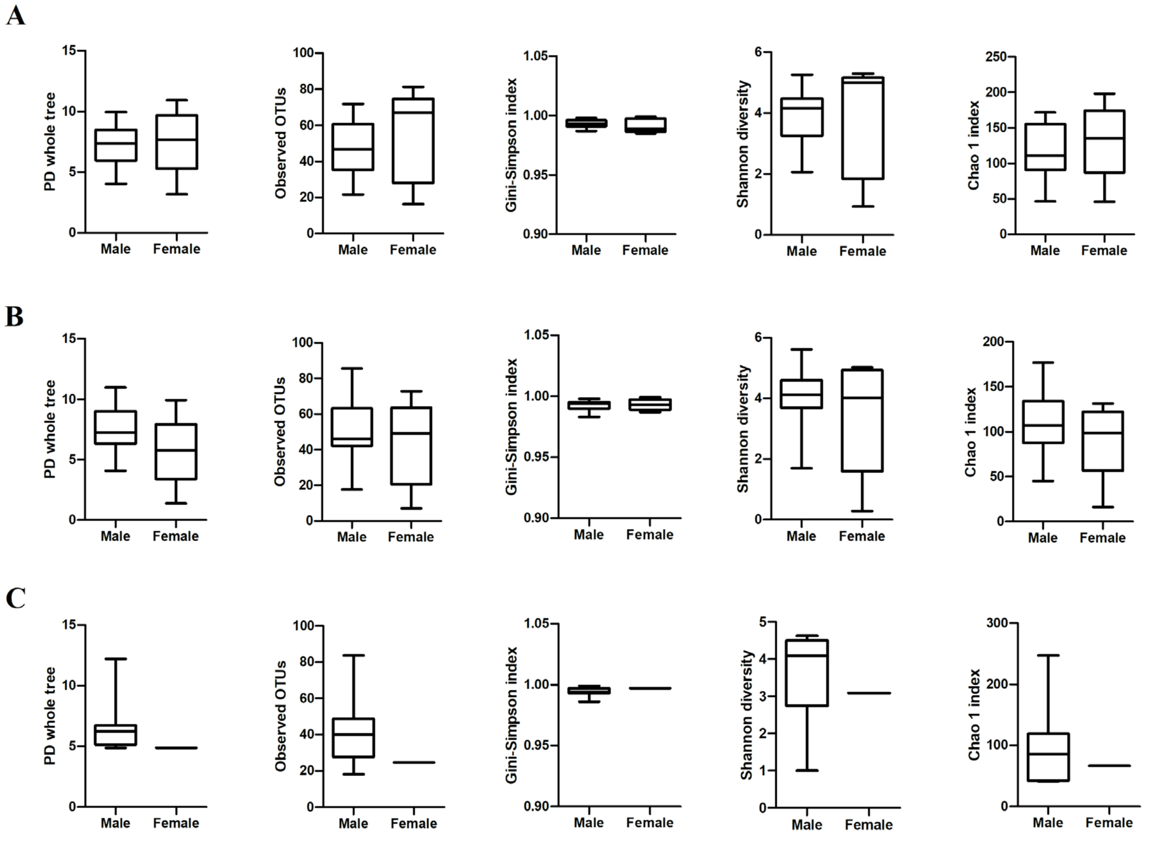

Figure S9. Different proportion of females and males does not result in differences in alpha diversity in bacterial communities (A) Alpha diversity (PD whole tree, Observed OTU´s, Gini-Simpson, Shannon and Chao1 index) of psoriatic in swabs of back samples. **(B)** Alpha diversity (PD whole tree, Observed OTU´s, Gini-Simpson, Shannon and Chao1 index) of psoriatic in scrappings of back samples. **(C)** Alpha diversity (PD whole tree, Observed OTU´s, Gini-Simpson, Shannon and Chao1 index) of psoriatic in biopsies of back samples. Statistical significance was confirmed using Mann-Whitney test. Significant differences are noted by *p < 0.05; **p < 0.01, and ***p < 0.001.
